# A Novel Furocoumarin Derivative, 5-((diethylamino)me-13 thyl)-3-phenyl-7H-furo [3,2-g] chromen-7-one Upregulates Melanin Synthesis via the Activation of cAMP/PKA and MAPKs Signal Pathway: In Vitro and In Vivo Study

**DOI:** 10.3390/ijms232214190

**Published:** 2022-11-16

**Authors:** Deng Zang, Chao Niu, Xueying Lu, Haji Akber Aisa

**Affiliations:** 1 State Key Laboratory Basis of Xinjiang Indigenous Medicinal Plants Resource Utilization, CAS Key Laboratory of Chemistry of Plant Resources in Arid Zone, Xinjiang Technical Institute of Physics and Chemistry, Chinese Academy of Sciences, Urumqi 830011, China; 2 University of Chinese Academy of Sciences, Beijing 100049, China

**Keywords:** furocoumarin derivatives, melanogenesis, cAMP/PKA and MAPK signaling

## Abstract

Psoralen, a major furocoumarin component of the Fructus Psoralen (FP), in combination with ultraviolet radiation, cures abnormal pigmentation disorder. In a previous study, we synthesized a series of linear furocoumarins with different substituents, out of which 5-((diethylamino)methyl)-3-phenyl-7H-furo [3,2-g] chromen-7-one (encoded as 5D3PC) showed better pigmenting effect than others in B16 cells. In this study, we examined the mechanism underlying the melanogenic effect of 5D3PC both in vivo and in vitro. To examine the pigmentation effect, the B16 and human melanocyte cell lines, PIG1 and PIG3V melanocytes were incubated with 5D3PC. In animal experiments, C57BL/6 mice received 5% hydroquinone and were administrated with 5D3PC for 30 days. 5D3PC upregulated the melanin synthesis and tyrosinase in B16 cell, PIG1 and PIG3V. The expression level of tyrosinase (TYR), tyrosinase-related protein-1 (TRP-1) and tyrosinase-related protein-2 (TRP-2), microphthalmia-associated transcription factor (MITF), cyclic adenosine monophosphate (cAMP), phosphorylation of cAMP-responsive element binding protein (p-CREB), phosphorylation of p38 mitogen-activated protein kinase (MAPK), c- phosphorylation of Jun N-terminal kinase (p-JNK) was significantly higher in 5D3PC-treated B16 cells. The oral administration of 5D3PC attenuated the depigmentation of the C57BL/6 vitiligo mice model by increasing the numbers of melanin-containing hair follicles, melanogenic protein, and melanogenesis-relative genes expression in skin tissues.

## 1. Introduction

Melanin synthesis plays a critical role in pigmentation disorders, which results in altered pigmentation of the skin, such as vitiligo, which is one of the most common pigmentation disorders [1]. Melanogenesis is a complex biochemical process responsible for producing melanin [2] and is controlled by TYR, TRP-1, and TRP-2, which are regulated by MITF, which mediates melanocyte proliferation and melanin production [3]. Transcriptional regulation of MITF via the activation of intracellular signaling is critical for inducing melanogenesis [4,5,6], while cAMP is an important factor for melanogenesis [7]. The cAMP molecule activates the cAMP/PKA pathway and induces CREB, which upregulates MITF transcript, and enhances melanogenesis [8]. MAPK pathway, including p38 MAPK, extracellular signal-regulated kinase (ERK), and JNK, is also reported to regulate MITF; and, therefore, could cause melanogenesis [9].

**Figure 1:**
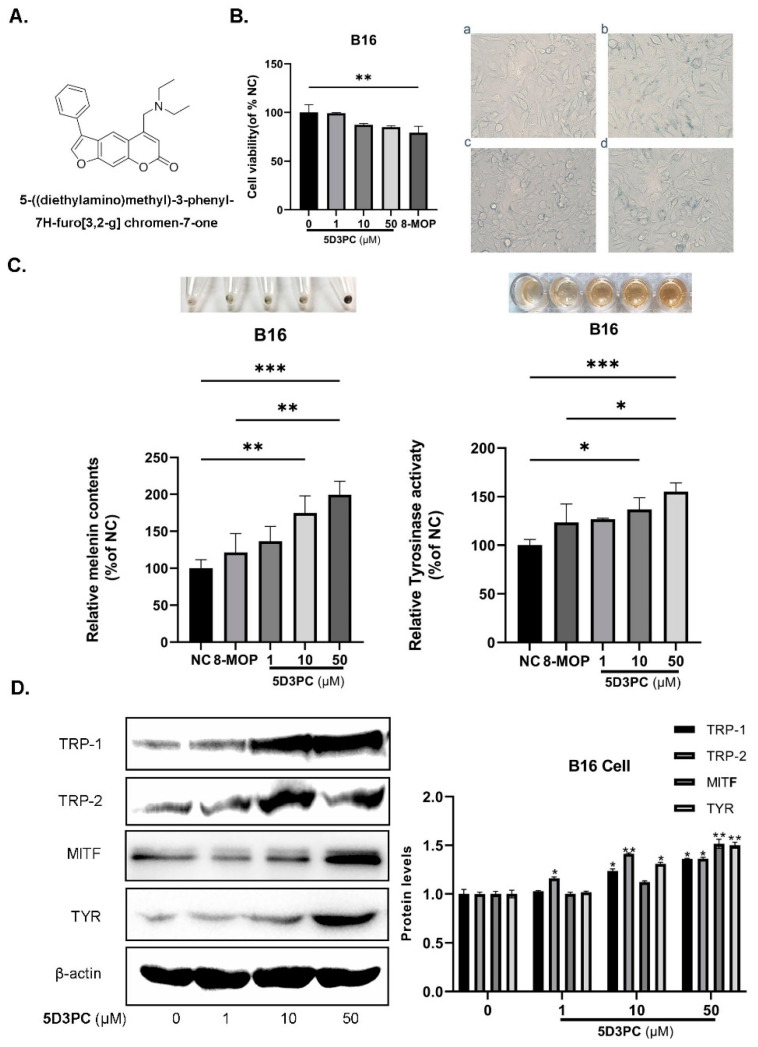
5D3PC promoted melanogenesis in B16 cells (**A**) Chemical structure of the 5D3PC; (**B**) Different concentrations (0–50 μM) of 5D3PC on B16 cell viability were measured by CCK-8 assay. Morphological changes in 5D3PC treated B16 cells at 200× magnification. (**a**) 0.1%DMSO (**b**) 1 µM 5D3PC, (**c**) 10 µM 5D3PC, and (**d**) 50 µM 5D3PC. (**C**) Effect of 5D3PC on melanin content and TYR activity in B16 cells. Melanin content of B16 cells treated with different concentrations (1, 10, and 50 µM) of 5D3PC for 48 h. TYR activity in the similarly treated B16 melanoma cells. DMSO (0.1%) was used as the vehicle control, and 8-MOP (50 µM) as the positive control. (**D**) Western blotting assays determined the levels of melanogenic genes in B16 cells treated with different concentrations (0–50 µM) of 5D3PC. All results are expressed as mean ± SD; statistical analysis was performed with one-way ANOVA, followed by Tukey’s multiple comparisons test. * *p* < 0.05, ** *p* < 0.01, and *** *p* < 0.001 compared with NC (negative control) group.

8-methoxypsoralen(8-MOP) is a well-known Furocoumarin component used with ultraviolet radiation (PUVA)in pigmentation disorder therapies [10]. Although 8-MOP has been shown to promote melanogenesis in a C57BL/6 vitiligo mice model previously [11], its mechanism of melanogenic effect remained uncertain. Several side effects such as skin cancer and hepatic steatosis have been reported with 8-MOP [12]. A number of experiments demonstrated that 8-MOP could reduce birth rates in rats and might have potential risk for infertility and/or birth defects and hepatotoxicity risk in a mouse [13]. Recently, we synthesized a new series of furocoumarin derivatives and evaluated their melanogenic effects in B16 murine cells, of which 5-((diethylamino)methyl)-3-phenyl-7H-furo [3,2-g] chromen-7-one (encoded as 5D3PC) (Figure 1A) showed a remarkable melanogenic effect in B16 murine cells. In this study, we found that 5D3PC could promote melanogenesis by cAMP/PKA and MAPK signal pathways in B16 cells. We further investigated the 5D3PC melanogenic effect and its mechanism using a hydroquinone-induced vitiligo mice model, as well as in human epidermal melanocytes cell lines, PIG1 and PIG3V. No other acute or sub-acute toxicities were observed in 5D3PC using the ICR mice. 5D3PC may be a potential therapeutic drug for the treatment of pigmentation disorders.

## 2. Results

### 2.1. The Effect of 5D3PC on Cell Viability and the Melanogenic Effect

**Figure 2:**
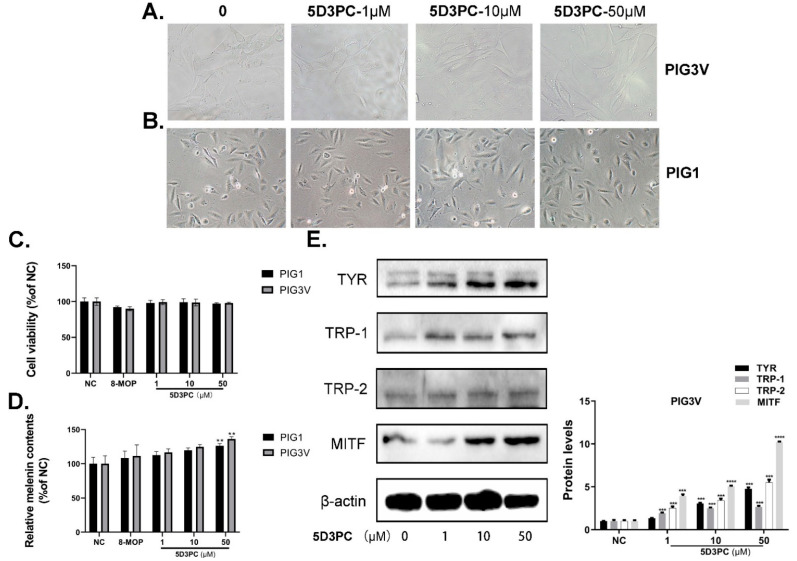
5D3PC promoted melanogenesis in human melanocytes; PIG1 and PIG3V. (**A**,**B**) Morphological changes in 5D3PC treated PIG3V cells at 200× magnification. (**C**) Different concentrations (0–50 μM) of 5D3PC on PIG3V and PIG1 cell viability were measured by CCK-8 assay. (**D**) Effect of 5D3PC on Melanin content of PIG3V and PIG1 melanocytes treated with different concentrations (1, 10, and 50 µM) of 5D3PC for 48 h. DMSO (0.1%) was used as the vehicle control, and 8-MOP (100 µM) as the positive control. (**E**) The expression of melanogenic genes in PIG3V cells treated with different concentrations (0–50 µM) of 5D3PC. All results are expressed as mean ± SD; statistical analysis was performed with one-way ANOVA, followed by Tukey’s multiple comparisons test. ** *p* < 0.01, *** *p* < 0.001 and **** *p* < 0.0001 compared with NC (negative control) group.

We used the CCK-8 cell viability assay to evaluate the cytotoxic effects of 5D3PC on B16 and PIG3V cells. Although B16 and PIG3V cells were treated with various concentrations of 5D3PC (0–50 µM), they did not show obvious cytotoxic effects or increase in cell number (Figure 1B and Figure 2A,B). Next, we evaluated the melanin contents and tyrosinase activity of 5D3PC to determine the melanogenic effect of 5D3PC. As shown in Figure 1C and Figure 2D, the 5D3PC not only significantly increased the cellular melanin contents and cellular TYR activity (*p* < 0.001) at concentrations of 10 µM and 50 µM.

### 2.2. 5D3PC Promotes the Expression of MITF and TRPs in B16 and PIG3V Cells

We evaluated the expressions of TYR, TRP-1, TRP-2, and MITF, which act as transcription regulators during the melanogenesis process. 5D3PC treatment showed a significant increase at concentrations of 10 µM and 50 µM in the expression of TYR, TRP-1, and TRP-2, which indicated that the 5D3PC could stimulate melanogenesis by enhancing the expression of these proteins. Additionally, 5D3PC could also increase the expression of TYR and MITF in B16 and PIG3V cells. These results indicated that 5D3PC could induce melanin biosynthesis in B16 and PIG3V cells by enhancing the expression of Tyrosinase and the MITF gene (Figure 1D and Figure 2E).

### 2.3. 5D3PC Ameliorates C57BL/6 Mice Model of Hydroquinone-Induced Vitiligo

**Figure 3:**
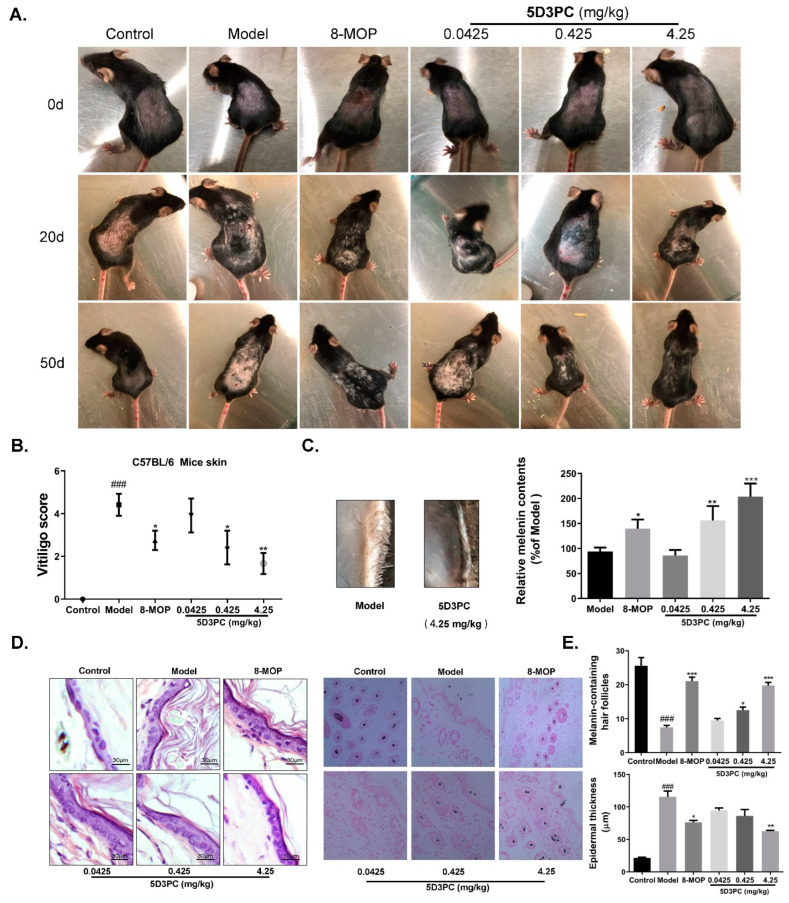
5D3PC ameliorates C57BL/6 mice model of hydroquinone-induced vitiligo. (**A**) Effect of 5D3PC on C57BL/6 mouse model; presentation of phenotype of mice from control, hydroquinone, and 5D3PC groups, respectively. Photograph is taken after 0 days of experiments, 20 days after hydroquinone induce model, and after 30 days of treatment. (**B**) The degree of depigmentation was scored blindly after 30 days. (**C**). Melanin content in skin tissue treated with different concentrations (0.0425, 0.425, and 4.25 mg/kg) 5D3PC. (**D**).H&E staining of C57BL/6 mice skin oral administration with vehicle, 8-MOP(4.25 mg/kg) or 5D3PC (0.0425 mg/kg, 0.425 mg/kg, 4.25 mg/kg). (**E**) the numbers of melanin-containing hair follicles in mouse dorsal skin by Lillie-stained. Quantification of melanin containing-hair follicles. All results are expressed as mean ± SD; statistical analysis was performed with one-way ANOVA, followed by Tukey’s multiple comparisons test. ^###^ *p* < 0.001 compare with control group. * *p* < 0.05, ** *p* < 0.01, and *** *p* < 0.001 compared with Model group.

C57BL/6 hair contained more melanin particles owing to its black color [14,15]. HQ induced depigmentation in C57BL/6 mice through tyrosinase inhibiting effect that prevented the tyrosinase-mediated conversion of DOPA to melanin [16], which also caused melanocyte and melanosome degradations [17]. Consequently, we choose the HQ-induce C57BL/6 mouse model to verify the effect of 5D3PC on melanogenesis. HQ model mice showed noticeable whitening of the dorsal skin and hair on the 50th day of the experiment. In contrast, the oral administration of 5D3PC for 30 days showed a progressive darkening of the dorsal skin and hair as compared with the HQ model group (Figure 3A). The extent of depigmentation was objectively quantified by an observer blinded to the experimental groups, using a point scale based on the extent of depigmentation at dorsal locations; as described previously, the dorsal location was examined, and the extent of depigmentation estimated as a percentage of the anatomic site [18]. Points were awarded as follows: no evidence of depigmentation (0%) received a score of 0, >0–10% = 1 point, >10–25% = 2 points, >25–75% = 3 points, >75–<100% = 4 points, 100% = 5 points. The Vitiligo Score of an experimental group is reported as the average individual score of each mouse within that group. The degree of depigmentation was scored 30 days after treatment; the clinically apparent skin darkening was most significant in the 4.25 mg/kg group (Figure 3B). Melanin content of the mice (skin) that received 5D3PC increased significantly as compared with the HQ model group, which was consistent with the magnitude of change seen in vitro (Figure 3C). The skin of C57BL/6 mice that received 5% HQ exhibited significant pathological injury and epidermal stratum corneum thickening, as shown in H&E staining. In contrast, the 5D3PC administration significantly reversed this phenomenon (Figure 3D).

### 2.4. The 5D3PC Administration Increases the Numbers of Melanin-Containing Hair Follicles

To further evaluate the 5D3PC-mediated melanogenic effect in our study, we collected the dorsal skin of mice and counted the number of melanin-containing hair follicles. The melanin of Hair follicles was almost invisible and completely absent in the test area of C57BL/6 mice (skin) that received 5% HQ, as described in Figure 3E. In contrast, the 5D3PC treatment increased the melanin content of hair follicles. One hundred cells were observed under high magnification (×200) (Olympus Optical Co., Ltd., Tokyo, Japan). The number of melanin-containing Hair follicles were counted.

### 2.5. The 5D3PC Treatment Enhances the Expression of TYR and TRP-1 in the Skin of C57BL/6 Mice

**Figure 4:**
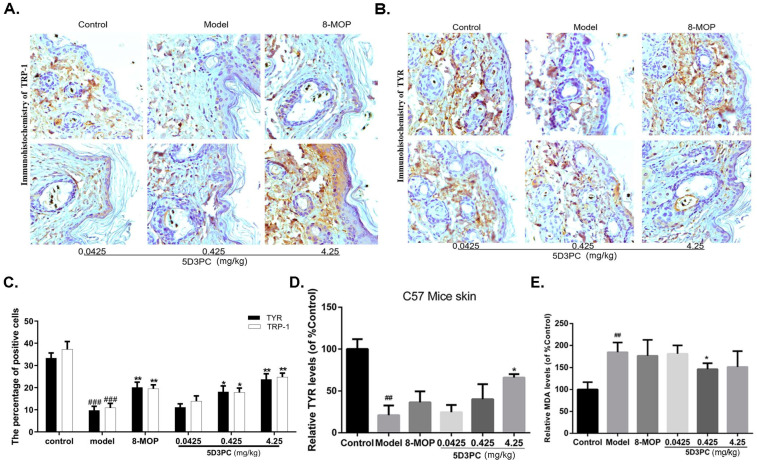
5D3PC enhances the expression of TYR and TRP-1 in the skin of C57BL/6 mice. (**A**) and (**B**) Effect of 5D3PC on expression and content of melanogenic protein in C57BL/6 mouse skin. Immunohistochemical staining of TYR and TRP-1 in the skin of mice. showed positive staining for TYR and TRP-1. (×200, *n* = 10). (**C**) Depict quantitative data of positive cells. (**D**) C57BL/6 mouse skin-TYR levels were analyzed using a TYR assay ELISA kit. (**E**) Effects of 5D3PC on content of malondialdehyde (MDA) in serum of mice (*n* = 10). C57BL/6 mouse serum-MDA levels were analyzed using an MDA assay ELISA kit. All results are expressed as mean ± SD; statistical analysis was performed with one-way ANOVA, followed by Tukey’s multiple comparisons test. ^##^ *p* < 0.01, ^###^ *p* < 0.001 compare with control group. * *p* < 0.05 and ** *p* < 0.01 compared with Model group.

To further explore molecular mechanisms of the 5D3PC-mediated reversal of HQ-induced vitiligo, the expressions of important regulators of melanogenesis (TYR and TRP-1) were measured by ELISA and immunohistochemical analysis. In our result, the expression levels of TYR and TRP-1 sharply increased in the test area of the skin, as shown in immunohistochemical analysis (Figure 4A–C), and the increase in the TYR expression was confirmed by the ELISA method (Figure 4D).

### 2.6. The 5D3PC Administration Reduces the Content of MDA in Serum

HQ induced depigmentation via melano-cytotoxicity, which destroyed melanocyte and melanosome degradation [19]. Hence, we studied the intervention effect of 5D3PC on HQ-induced melano-cytotoxicity. Our result demonstrated that HQ could increase MDA levels in serum; however, 5D3PC levels reduced the production of MDA in a concentration-dependent manner (Figure 4E).

### 2.7. 5D3PC Induces Melanogenesis by Activating the cAMP/PKA Signal Pathway

**Figure 5:**
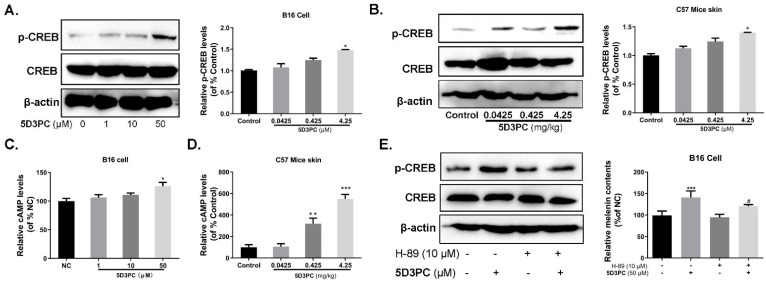
5D3PC induces melanogenesis by activating the cAMP/PKA signal pathway. (**A**) The levels of p-CREB in B16 cells were determined by WB analysis. (**B**) the levels of p-CREB after being treated with 5D3PC in C57BL/6 mouse model. p-CREB proteins were analyzed relative to β-actin expression. (**C**) B16-intracellular cAMP levels and (**D**) C57BL/6 mouse skin-cAMP levels were analyzed using a cAMP assay ELISA kit. (**E**) 5D3PC-induced melanin synthesis and p-CREB expression were blocked by PKA inhibitors in B16 cells. All results are expressed as mean ± SD; statistical analysis was performed with one-way ANOVA, followed by Tukey’s multiple comparisons test. ^#^ *p* < 0.005, * *p* < 0.05, ** *p* < 0.01, and *** *p* < 0.001 compared with NC (negative control) group.

We evaluated the expression level of p-CREB and the concentration of cAMP. The results indicated an increase in p-CREB expression levels significantly with high concentrations of 5D3PC in B16 cells (Figure 5A). The intracellular cAMP level enhanced with 5D3PC at the dose of 50 µM (Figure 5C), while the 5D3PC-mediated melanogenic effect and p-CREB expression in B16 cells were abrogated by a PKA inhibitor H89 (Figure 5E). 5D3PC treatment is reported to raise cAMP levels in B16 cells (result above). Therefore, we further examined the effect of 5D3PC on cAMP in the skin of C57BL/6 mice. The dorsal skin-extracted proteins were subjected to ELISA analysis, and oral administration of 5D3PC consistently increased the cAMP levels in the skin epidermis (Figure 5D). Next examined whether 5D3PC could affect CREB in the skin of C57BL/6 mice. Oral administration with 5D3PC in vivo consistently enhanced CREB phosphorylation in the skin of C57BL6 mice (Figure 5B).

### 2.8. 5D3PC Activates p38MAPK and SAPK/JNK, but Not ERK MAPK

**Figure 6:**
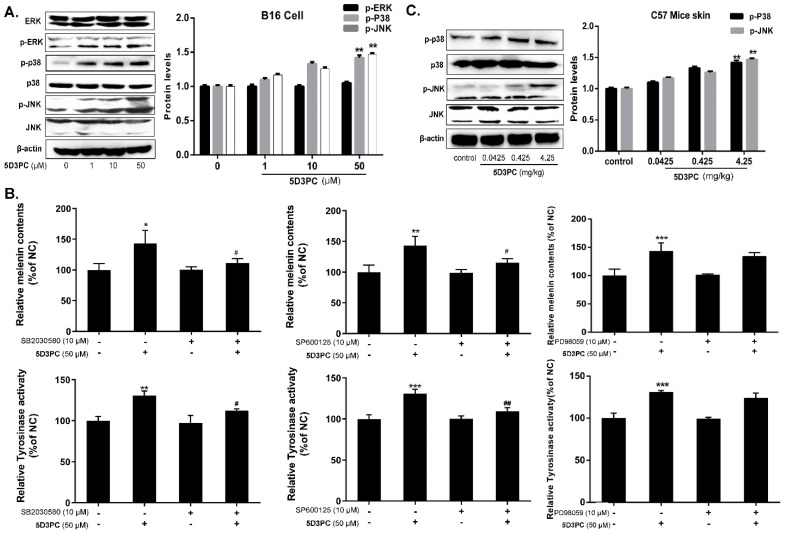
5D3PC activates p38MAPK and SAPK/JNK. (**A**) WB analysis for the expressions of p-P38, p-JNK, and p-ERK in B16 cells treated with different concentrations (0-50 µM) of 5D3PC. (**B**) B16 cells were pretreated with SP600125 (a JNK inhibitor), SB203580 (a p38MAPK inhibitor), and ERK inhibitor (PD98059) for 2 h and then incubated with 5D3PC (50 µM) for 48 h to measure the melanin contents and 24 h to measure TYR activity. (**C**) The levels of p-P38 and p-JNK were determined by WB analysis in C57BL/6 mouse model treated with 5D3PC. All results are expressed as mean ± SD; statistical analysis was performed with one-way ANOVA, followed by Tukey’s multiple comparisons test. * *p* < 0.05, ** *p* < 0.01, *** *p* < 0.001. ^#^ *p* < 0.05, and ^##^ *p* < 0.01 compared with only treated with 5D3PC.

Previous research has confirmed that MAPK signaling pathways were involved in regulating melanogenesis. In this study, we examined the influence of 5D3PC on the activation of the MAPK signal pathway. Our result showed that treated with 5D3PC could induce phosphorylation of P38 MAPK and JNK in B16 cells at 50 μM. However, 5D3PC did not enhance the expression of ERK (Figure 6A). We found out that the p-p38, p-jnk expression, and melanogenic effect (melanin content and tyrosinase activity) induced by 5D3PC in B16 cells was reduced by the SP600125 (a JNK inhibitor) and the SB20358 (a p38MAPK inhibitor) (Figure 6B). Next, we examined the effect of 5D3PC on p38 and JNK in the skin of the C57BL/6 mice model. Oral administration of mice with 5D3PC in vivo consistently enhanced P38 MAPK and JNK phosphorylation in the skin of the C57BL6 mice model (Figure 6C).

### 2.9. Toxicity Study of 5D3PC

**Figure 7:**
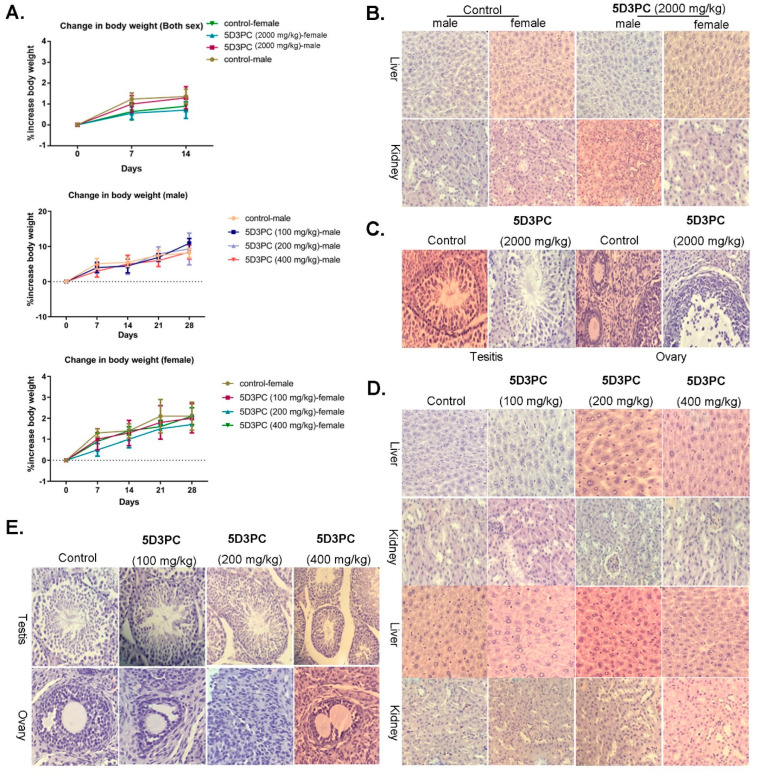
5D3PC did not show acute or sub-acute toxicities. (**A**) Body weight of control and 5D3PC administered male and female mice. 5D3PC treatment has no major impact on body weights of acute study. (**B**) Photomicrography of liver, kidney, testis, and ovary of acute study. (×200, *n* = 2). (**C**) 5D3PC treatment has no major impact on body weights of sub-acute study. (**D**) Photomicrography of liver, kidney (**E**) testis, ovary in control and 5D3PC administration mice of acute and sub-acute study. (×200, *n* = 5). All results are expressed as mean ± SD.

The administration of a single oral dose of 5D3PC at the dose of 2000 mg/kg (14 days observation period) or repeated dose at 100 mg/kg, 200 mg/kg, 400 mg/kg (28 days observation period) did not show any noticeable sign of toxicity in ICR mice. There was a significant increase in the weight of mice in both acute and subacute studies throughout the experiments among all treatment groups. However, there was no significant difference in the body weight gain between the control and treated groups (Figure 7A). The relative organ weight and food intake were not affected by 5D3PC. Furthermore, the hematological evaluation and the biochemical analysis showed that (Table 1 and Table 2) the neutrophil level increased in male mice (2000 mg/kg), and decreased in the female (2000 mg/kg) and male mice (100, 200 mg/kg) as compared to the control group, the eosinophils level increased in both sexes of all experimental group when compared to the control group, the monocytes level increased in all experimental group except male mice (200 mg/kg) and female mice (2000 mg/kg) when compared with the control group. The lymphocyte level decreased in male mice (200, 400 mg/kg) as compared to the control group. However, a decrease in CRE (all treatment group), a slight increase in AST (400, 2000 mg/kg), URE (all treatment group) in both sexes, and no significant differences in the other biochemical parameters were observed. Following the histopathological examination of the kidney, intact normal proximal and distal convoluted tubules were observed in the male and female mice of the control group, which were also identified in the 5D3PC group. The normal sinus hepaticus structures were observed in both the treatment and control group mice liver, but the number of corpus luteum (CL) and growing follicles in the ovary were not significantly different than those identified in the control group. The number of spermatogonia, primary spermatocytes, spermatids, and sperms did not show significant changes in testis histopathological analysis of all experimental groups as compared to the control group (Figure 7B–E).

## 3. Discussion

The aberrant loss and destruction of melanin pigments cause skin disorders with depigmented patches, such as vitiligo [20]. Although several melanogenic agents have been developed, most of them have a potential risk of side effects. Psoralen is a major Furocoumarin component of the Fructus Psoraleae [21], which is used with ultraviolet radiation for the treatment of abnormal pigmentation disorder. Recently, we synthesized a series of linear furocoumarins with different substituents, of which 5-((diethylamino)methyl)-3-phenyl-7H-furo [3,2-g] chromen-7-one (encoded as 5D3PC) showed a remarkable melanogenic effect in B16 murine cells. In this study, we investigated the melanogenic effect of 5D3PC in vitro and in vivo. Melanin synthesis is catalyzed by three structurally related proteins, TYR, TRP-1, and TRP-2 [22], which are transcriptionally regulated by MITF that acts as a master regulator for skin pigmentation [23]. In our study, we observed that 5D3PC derivatives could significantly increase tyrosinase activity and melanin content at concentrations of 10 µM and 50 µM in B16 cell, human melanocyte cell line (PIG1), and vitiligo melanocyte cell line (PIG3V). 5D3PC promoted the expression level of melanogenic enzymes (TYR, TRP-1, TRP-2) and MITF at concentrations of 10 µM and 50 µM in B16 cells and enhance the expression level of TYR, and MITF at concentrations of 10 µM and 50 µM in PIG3V cells. Melanogenesis is an extremely complex cascade reaction, and more than 255 genes are involved in melanogenesis via the regulation of MITF and TYR expression [24]. The MAPK pathway plays a critical role in melanogenesis and is modulated by the phosphorylation of p38 and JNK [25]. Our result showed that the 50 µM 5D3PC treated B16 cells significantly promoted the phosphorylation of p38 and JNK with the exception of ERK phosphorylation. The p38 and JNK inhibitors blocked the effect of the 5D3PC-induced melanin synthesis process in B16 cells. The activation of the cAMP/PKA signaling pathway increased cAMP levels and activated PKA, and thereafter, the phosphorylation of the CREB transcription factor. CREB upregulated the transcription of CREB’s target genes MITF in melanocytes [26]. In our study, the expression level of p-CREB increased, and the intracellular cAMP concentration also enhanced at concentrations of 50 µM, while the 5D3PC mediated melanogenic effect in B16 cells was almost completely blocked by H89, a PKA inhibitor. The role of 5D3PC as a potential drug for the treatment of hypopigmentation disorder was verified in an animal model. HQ-induced depigmentation in C57BL/6 mice recapitulated the characteristic of human hypopigmentation disorder (i.e., Vitiligo), which is characterized by the loss of functional melanocytes from the hair follicles. Therefore, we chose this model to study the effect of 5D3PC on melanogenesis. On the 30th day of 5D3PC treatment, the HQ model mice showed obvious whitening of the dorsal skin and hair. In contrast, the 5D3PC-treated mice showed a progressive darkening of the dorsal skin and hair. The epidermal stratum corneum thickened in the HQ-tested area of the skin, which suggested that this phenomenon was similar to human vitiligo. Our results corroborated with studies reported by Stern and co-workers [27], which showed that the thick stratum corneum in vitiligo compensated for the absence of pigmentation [28,29]. In contrast, the 5D3PC could significantly reverse this phenomenon which indicated that it could also reverse the epidermal stratum corneum thickening caused by HQ. We analyzed the distribution of melanin particles in the skin and hair follicles of mice by the Lillie staining method, which indicated that the melanin was completely absent in the hair follicles of mice treated with HQ. However, the 5D3PC administration significantly enhanced the melanin content of hair follicles. Tyrosinase transcription, translation, and enzyme activity highly regulated melanogenesis during the development of murine hair follicle that reflected the skin color in C57BL/6 mice [30]. HQ induced depigmentation in C57BL/6 mice through its tyrosinase inhibiting effect; therefore, the effect of 5D3PC on the regulation of tyrosinase gene expression in C57BL/6 mice skin was evaluated by Tyrosinase expression through ELISA and immunohistochemical analysis. Our results revealed that the expression of TYR and TRP-1 significantly increased in the HQ and 5D3PC-treated group as compared with the HQ model group. C57BL6 mice skin was used to further validate the role of cAMP/PKA, P38 MAPK, and JNK signal pathways in the 5D3PC-induced synthesis of melanin. HQ is a well-known melanocyte-toxic chemical that oxidizes melanocytes and is responsible for the destruction of pigment cells that leads to skin depigmentation [31]. As MDA is a key marker of lipid peroxidation, the progression of melanocyte damage has been correlated with oxidative stress [32]. In our study, we found out that HQ increased MDA in serum, whereas the 5D3PC reduced the production of MDA in a concentration-dependent manner which indicated that 5D3PC is capable of protecting melanocyte of skin against melanocyte-toxic hydroquinone. We further evaluated the toxicological parameters of our therapeutic intervention (5D3PC) before using it as a vitiligo therapeutic drug. We evaluated the single dose (2000 mg/kg) acute study and the repeat dose (100, 200, and 400 mg/kg) sub-acute study to identify the toxicity levels. No morbidity and mortality were observed in both acute or sub-acute studies. The LD50 value was estimated to be in the range of 2000–5000 mg/kg, included in category 5 (OECD 423, 2001). We evaluated the probable organ toxicity of two main organs responsible for drug metabolism and excretion. The hepatotoxicity was evaluated by measuring ALT and AST, and the renal function was accessed by quantifying CRE and URE. Our results indicated a prominent change in biochemical and hematological parameters; however, it was within the normal range and could be attributed to individual differences among mice [33]. 8-MOP has been reported to have reproductive toxicity [34] and, therefore, was further evaluated for possible 5D3PC-induced reproductive toxicity in an ovary and testis. In our histological examination, the intact ovary and testis showed no signs of 5D3PC-induced toxicity.

## 4. Materials and Methods

### 4.1. Drug and Materials

The 5D3PC was synthesized by the Key Laboratory of Plant Resource Sand Chemistry of Arid Zone, Xinjiang Technical Institute of Physics and Chemistry, Chinese Academy of Sciences. Yield 43%, a light yellow solid, m. pt. 138–140 °C; 1H NMR (400 MHz, CDCl3), δ: 8.42 (s, 1H), 7.84 (s, 1H), 7.66 (d, J = 7.4 Hz, 2H), 7.55–7.48 (m, 3H), 7.42 (t, J = 7.2 Hz, 1H), 6.58 (s, 1H), 3.77 (s, 2H), 2.61 (q, J = 7.0 Hz, 4H), 1.09 (t, J = 7.0 Hz, 6H); 13C NMR (101 MHz, CDCl3), δ: 161.50, 157.12, 152.18, 142.87, 131.30, 129.31, 128.14, 127.67, 123.82, 122.47, 116.67, 115.79, 113.77, 100.22, 55.36, 47.79, 12.08; IR (KBr), 𝜈/cm^−1^: 2926, 1725, 1635, 1574, 1456, 1385, 1318, 1260, 1113, 1042, 985, 867; HRMS (ESI), m/z calcd. for C22H22NO3[M + H] +: 348.1594; found: 348.1680. The synthesis and analysis procedures were described by Niu et al. [35]. 5D3PC dissolved in DMSO was stored at −20 °C as a stock solution (50 mM). L-3-(3,4-Dihydroxyphenyl) and alanine (L-DOPA) (CAS:59-92-7) were purchased from Generay Biotech (Shanghai, China), and 8-Methoxypsoralen (8-MOP) (CAS:298-81 -7) from Sigma Aldrich (Milan, Italy). CREB (86B10), p-CREB (Ser133) (1B6), p38 (L53F8), p-p38 (Thr180/Tyr182) (28B10), SAPK/JNK (#9252s), p-JNK (Thr183/Tyr185), ERK(L34F12), and p-ERK(Thr202/Tyr204) (E10) were purchased from Cell Signaling Technology (Danvers, MA, USA). Antibodies against β-actin (8H10D10) and tyrosinase (C-19), TRP-1 (H-90), and TRP-2 (H-150) were purchased from Santa Cruz Biotechnology, Inc. (Santa Cruz, CA, USA). Anti-MITF antibody was obtained from Chemicon (Temecula, CA, USA). Goat anti-rabbit (BA1054), goat anti-mouse (BA1050), rabbit anti-goat (BA1060) antibodies were obtained from BOSTER Biological Technology (Wuhan, China). SP600125(CAS:129-56-6), SB203580 (CAS:152121-47-6) H89 (CAS:127243-85-0) were obtained from Beyotime Biotechnology (Shanghai, China).

### 4.2. Cell Culture

Experiments were conducted in B16 cells (B16, Cat# TCM2), which were cultured in DMEM (Gibco Life Technologies, Waltham, MA, USA) supplemented with 10% FBS (Thermo Fisher Scientific), penicillin G (100 U/mL), and streptomycin (100 µg/mL) (Gibco-BRL, Grand Island, NY, USA), incubated at 37 °C in an atmosphere containing 5% CO2. PIG1 (immortalized normal human melanocyte cell line) and PIG3V (a vitiligo melanocyte cell line) provided by Dr. Caroline Le Poole (Loyola University Chicago, Maywood, IL, USA), both maintained in Medium 254 with Human Melanocyte Growth Supplement (Gibco Life Technologies, Waltham, MA, USA), 5% FBS (Gibco Life Technologies, Waltham, MA, USA) and penicillin G (100 U/mL), and streptomycin (100 µg/mL) at 37 °C in the presence of 5% CO_2_.

### 4.3. Cell Viability Assay

Cells seeded at a density of 5 × 10^3^ cells/well in 96-well were incubated with 5D3PC for 24 h. Then the culture medium was replaced with the CCK-8 (Absin, Shanghai, China) solution (10 µL), and the cells were further cultured for 2 h at 37 °C. Plates were read at 450 nm using a microplate reader Spectra Max M5 (Molecular Devices company, San Diego, CA, USA). An equal volume of cells without the treatment was used as a blank control. All the experiments were repeated three times.

### 4.4. Tyrosinase Activity

The cellular tyrosinase activity was estimated by measuring the rate of L-3, 4dihydroxyphenylalanine (L-DOPA) oxidase activity [36,37]. Briefly, the process involved culturing the B16 cells plated on six-well plates (3.5 × 10^5^ cells per well) and treated with 5D3PC for 24 h at 37 °C. The cells were then washed with cold PBS and lysed in a PBS buffer containing 1% TritonX-100 + 1% sodium deoxycholate. The cell lysates were centrifuged at 12,000× *g* for 20 min, and 90 µL of this supernatant and 10 µL of 10 mM L-DOPA solution was mixed and plated in 96-well plates and incubated at 37 °C for 30 min. The optical densities were measured at 490 nm using a microplate reader.

### 4.5. Melanin Contents Measurement

The melanin contents were measured using a previously described method with a slight modification [38]. Briefly, the B16 and PIG3V cells plated on six-well plates (2 × 10^5^ cells per well) were incubated with various (1–50 µM) concentrations of 5D3PC for 48 h at 37 °C. Following this, the cells were lysed in RIPA Lysis buffer (AR0105-100) (BOSTER Biological Technology, Wuhan, China) and centrifuged at 12,000× *g* for 20 min. A total of 3 µL supernatants of the cell extracts were used to measure the total protein content by the BCA kit assay (PP02) (Biomed, Beijing, China). Then discarding the remaining part, 190 µL 1 mM NaOH was added at 80 °C for 1 h. The optical density of the supernatant was measured at 405 nm. For the tissue melanin assays, as with previous reports [39], tissues were weighed prior to boiling in 1 M NaOH for 1 h and centrifuged at 12,000 rpm for 20 min discarding the insoluble materials. Following this, the optical density of the supernatants was measured at 405 nm and normalized to the weight of the tissue.

### 4.6. Western Blot Analysis

The protein of the B16 and PIG3Vcells was prepared as mentioned in the previous section above. For the C57BL/6 mice, dorsal skin was lysed in 450 µL lysate buffer (P0013) (Beyotime Biotechnology, Shanghai, China) using the Tissue Lyser LT (Qiagen Hilden Germany). All proteins per lane were separated by 10% SDS-PAGE, transferred to PVDF membrane, blocked with 5% skim milk or 5% BSA, and exposed overnight at 4 °C with appropriate antibodies. Following incubation with the second antibody, the protein bands were detected using an ECL (enhanced chemiluminescence) Western blotting detection kit and quantified with a Chemi Doc MP Imaging system (Bio-Rad Laboratories, Inc., Hercules, CA, USA). All experiments were performed three times.

### 4.7. cAMP Measurement in the B16 Cells

The cAMP concentration was measured using a cAMP ELISA kit (Cell, Biolabs, Inc., San Diego, CA, USA) according to the manufacturer’s protocols; absorbance of all samples was measured at 450 nm using a microplate reader.

### 4.8. Animals

C57BL/6 mice (20 ± 2 g, 4–5 weeks) were purchased from the Vital River Laboratory Animal Technology Co., Ltd., Beijing, China (Approval ID: SCXK-(jing) 2021-011). For the vitiligo animal model, ICR mice (4–5 weeks) were purchased from Xinjiang Medical University (Approval ID: SCXK-(xin) 2018-0002. All experimental animals were maintained in a humid (50 ± 10%) temperature (22 ± 4 °C). Experiments were performed after formal approval by the Institutional Ethical Committee for the laboratory of Xinjiang Medical University (Approval ID: SYXK-(xin) 2018-0003.

### 4.9. Experimental Animal Design and Drug Administration

As previously described [40], C57BL/6 mice were randomly divided into six groups: (1) Control group (*n* = 10): distilled water was administrated and smeared. (2) Hydroquinone model group (*n* = 10): received 5% Hydroquinone (HQ) (Jiang lai) on the 2 × 2 cm shaved dorsum for 50 days and administrated with distilled water for 30 days. (3) 8-MOP group (*n* = 10) received 5% hydroquinone for 50 days and was administrated with 8-MOP (4.25 mg/kg) for 30 days. (4)–(6) the 5D3PC group: received 5% hydroquinone for 50 days and administrated with 5D3PC at various concentrations (0.0425 mg/kg group (*n* = 10), 0.425 mg/kg group (*n* = 10), 4.25 mg/kg group (*n* = 10) for 30 days). An acute oral toxicity test was conducted as per the OECD guideline No. 423. [41], the single dose (2000 mg/kg) of 5D3PC was administered orally to both the male and female (*n* = 4) ICR mice. In the subacute study, the 5D3PC was given at doses of 100, 200, and 400 mg/kg by oral gavage to both the male and female (*n* = 5) ICR mice during 28 days as per the OECD guideline No. 407 [42]. All mice were observed individually during the first 30 min, first 24 h, and daily thereafter, acute and subacute studies totaling 14 and 28 days, respectively. Biochemical, hematological, and histological analyzes were performed.

### 4.10. Hematological Evaluation and Biochemical Analysis

For hematological analysis, white blood cell (WBC), % of lymphocytes, neutrophils, monocytes, eosinophils, basophils, red blood cell (RBC), hemoglobin, hematocrit, mean corpuscular hemoglobin (MCH), mean corpuscular volume (MCV) red blood cell amplitude (RDW) were performed in electronic counter (BC-2800 Vet-Auto Hematology Analyzer, Mindray, Shenzhen, China). As for the biochemical analysis, the enzymes aspartate aminotransferase (AST), alanine aminotransferase (ALT), urea (URE), and creatinine (CRE) analysis were performed in a semi-automatic analyzer (Bioclin Mindray BA 88A^®^).

### 4.11. Pathological Analysis

For the pharmacodynamic study, the skin was fixed with 4% paraformaldehyde and stained with hematoxylin-eosin (H&E) for identifying the skin structures and analysis. To further observe the melanin-containing epidermal cells and melanin-containing hair follicles, Lillie staining was performed and observed using Leica Microsystems (CMS GmbH DM6000B, Wetzlar, Germany). For the toxicity test, liver, kidney, testis, and ovary samples were fixed in 4% paraformaldehyde, stained in hematoxylin-eosin (H&E), and analyzed for the integrity of the tissue structures.

### 4.12. Immunohistochemistry Analysis

Before dewaxing, the tissue chip was placed overnight in a 70 °C constant temperature box immersed in dimethylbenzene and ethanol for dewaxing and hydration. The tissue sections were heated in a microwave with sodium citrate buffer (PH6.0) for 20 min at 95 °C. After cooling to 50 °C, the tissue chip was washed thrice in PBS and incubated with 50 μL of hydrogen peroxide solution (KIT-9730-A) for 10 min at room temperature to block the endogenous peroxidase and washed thrice with PBS. The sections were then incubated overnight at 4 °C in TYR (1:50 sc-20035) and TRP-1 (1:50 sc-166857) antibodies (Santa Cruz, CA, USA). The tissue chip was then incubated with a secondary antibody (Enzyme-labeled goat anti-rabbit IgG polymer PV-6001) for 1 h at room temperature and washed thrice with PBS. The DAB solution was applied for 5 min, washed in tap water, and counterstained with Hematoxylin Stain for 2 min. After the hydrochloric acid differentiation, a tissue chip was washed in tap water for 3 min, dehydrated, mounted in a mounting media and sealed with a cover slip, and examined under the microscope.

### 4.13. TYR, cAMP Content of Skin, and MDA Content in the Serum of the C57BL/6 Mice

The content of TYR, cAMP in the skin, and the content of malondialdehyde (MDA) in serum were performed using commercial kits according to the manufacturer’s protocols (Elabscience Biotechnology Co., Ltd., Wuhan, China). The absorbance of all samples was measured at 450 nm using a microplate reader.

### 4.14. Statistical Analysis

All results are expressed as mean ± SD; statistical analysis was performed with one-way ANOVA, followed by Tukey’s multiple comparisons test. Statistical analysis was performed using GraphPad Prism 6 (La Jolla, CA, USA). *p*-values < 0.05 were considered to be statistically significant.

## 5. Conclusions

In conclusion, 5D3PC promoted melanogenesis by upregulating melanin synthesis and tyrosinase in-vitro in the B16 cell, PIG1, and PIG3V cell line. Additionally, the oral administration of 5D3PC attenuated the depigmentation of hydroquinone-induced vitiligo via an increase in the number of skin basal layer melanocyte and melanin-containing hair follicles. The 5D3PC upregulated melanin synthesis and tyrosinase in a dose-dependent manner, which enhanced the expression of TYR, TRP-1, TRP-2, and MITF. Considering that 5D3PC increased expression of TYR, TRP-1,2, and MITF via the activation of cAMP/PKA and MAPK signal pathway leads to increased melanin synthesis and tyrosinase activity. However, 5D3PC did not show any acute or sub-acute toxicities. Although the HQ-induced vitiligo mouse model is an established model for evaluating melanogenesis, it reflected only certain aspects of pathogenesis as observed in vitiligo. Therefore, extensive research is necessary to evaluate melanogenesis in other vitiligo animal models, which can portray the clinical effects of vitiligo.

## Figures and Tables

**Table 1:** Effect of 5D3PC (2000 mg/kg) on hematological and biochemical profilin ICR mice from 14 days acute toxicity study.

Sex	Parameter	Control	5D3PC 9609042000 mg/kg
Male	WBC (×10^3^/µL)	0.220 ± 0.060	0.160 ± 0.060
Female	Lymphocytes (%)	91.55 ± 4.450	69.80 ± 4.380
	Neutrophils (%)	3.700 ± 0.380	11.00 ± 3.110
	Monocytes (%)	2.250 ± 0.070	16.25 ± 5.440
	Eosinophils (%)	2.500 ± 0.560	2.950 ± 0.210
	Basophilic (%)	0.000 ± 0.000	0.000 ± 0.000
	RBC (×106/µL)	3.550 ± 0.250	1.630 ± 0.170
	HBG (g/dL)	63.00 ± 4.450	29.50 ± 2.890
	HCT (%)	18.65 ± 1.320	7.950 ± 0.840
	MCV (f L)	52.50 ± 0.280	50.95 ± 3.600
	MCHC (g/dL)	338.5 ± 2.120	387.0 ± 43.84
	RDW (%)	14.65 ± 0.210	15.85 ± 0.210
	AST (U/L)	93.35 ± 4.310	158.2 ± 5.020
	ALT(U/L)	20.95 ± 0.210	45.30 ± 3.390
	CRE (µmol/L)	33.80 ± 1.270	10.31 ± 1.670
	URE (mg/dL)	6.770 ± 0.370	49.25 ± 4.030
	WBC (×103/µL)	1.950 ± 0.860	5.560 ± 0.320
	Lymphocytes (%)	78.05 ± 15.34	71.15 ± 1.760
	Neutrophils (%)	14.15 ± 11.50	22.90 ± 1.550
	Monocytes (%)	3.900 ± 0.390	3.000 ± 0.120
	Eosinophils (%)	3.550 ± 0.070	2.300 ± 0.110
	Basophilic (%)	0.000 ± 0.000	0.650 ± 0.070
	RBC (×106/µL)	7.990 ± 0.080	8.870 ± 0.700
	HBG (g/dL)	147.5 ± 4.300	168.5 ± 14.84
	HCT (%)	39.30 ± 7.350	45.60 ± 4.030
	MCV (f L)	49.15 ± 0.070	51.45 ± 0.490
	MCHC (g/dL)	372.0 ± 33.94	369.5 ± 0.700
	RDW (%)	14.90 ± 1.550	14.50 ± 0.000
	AST (U/L)	93.00 ±1.830	142.5 ± 2.260
	ALT (U/L)	21.35 ±1.060	42.80 ± 3.670
	CRE (µmol/L)	9.750 ± 0.160	9.030 ± 0.450
	URE (mg/dL)	7.040 ± 0.100	53.30 ± 3.390

^1^ 5D3PC did not show significant alteration in hematological and biochemical parameters in ICR in both sex mice. Values expressed as *n* ± SD. Data are considered statistically significant when *p* < 0.05.

**Table 2:** Effect of 5D3PC (2000 mg/kg) on hematological and biochemical profilin ICR mice from 14 days acute toxicity study.

Sex	Parameter	Control	5D3PC 100 mg/kg	5D3PC 200 mg/kg	5D3PC 400 mg/kg
Male	WBC (×10^3^/µL)	4.050 ± 0.280	2.070 ± 0.670	2.560 ± 1.390	2.660 ± 0.560
Female	Lymphocytes (%)	93.37 ± 3.940	80.95 ± 8.500	70.82 ± 3.430	69.05 ± 6.320
	Neutrophils (%)	20.27 ± 3.900	13.42 ± 0.830	13.82 ± 1.640	23.70 ± 5.260
	Monocytes (%)	11.95 ± 2.640	15.07 ± 4.240	11.97 ± 2.820	14.80 ± 1.980
	Eosinophils (%)	1.700 ± 0.530	5.370 ± 1.280	3.220 ± 1.260	2.250 ± 0.370
	Basophilic (%)	0.350 ± 0.050	0.420 ± 0.170	0.220 ± 0.090	0.200 ± 0.140
	RBC (×106/µL)	12.15 ± 0.430	6.650 ± 1.200	9.220 ± 1.370	9.150 ± 1.220
	HBG (g/dL)	194.3 ± 18.00	130.0 ± 12.40	120.0 ± 10.09	157.7 ± 19.63
	HCT (%)	63.87 ± 4.760	49.55 ± 1.930	48.72 ± 3.760	52.77 ± 9.33
	MCV (f L)	49.15 ± 2.160	50.35 ± 2.290	50.42 ± 1.260	49.55 ± 1.840
	MCHC (g/dL)	350.8 ± 11.32	344.5 ± 7.930	344.5 ± 3.100	351.5 ± 3.100
	RDW (%)	14.35 ± 1.410	15.87 ± 1.040	13.57 ± 0.850	13.87 ± 1.140
	AST (U/L)	96.93 ± 4.860	109.1 ± 6.730	109.8 ± 7.790	145.2 ± 11.86
	ALT (U/L)	25.53 ± 5.290	43.83 ± 3.630	41.05 ± 5.710	44.03 ± 4.500
	CRE (µmol/L)	36.03 ± 3.150	10.79 ± 0.810	11.52 ± 1.660	11.01 ± 1.550
	URE (mg/dL)	9.060 ± 2.950	47.39 ± 5.560	52.36 ± 1.720	50.06 ± 1.470
	WBC (×103/µL)	4.040 ± 0.180	3.030 ± 1.030	3.390 ± 0.420	4.140 ± 0.330
	Lymphocytes (%)	90.45 ± 1.070	90.60 ± 1.070	83.40 ± 4.290	85.30 ± 5.540
	Neutrophils (%)	23.12 ± 0.830	18.10 ± 1.280	17.92 ± 1.750	19.92 ± 1.770
	Monocytes (%)	10.76 ± 1.070	19.55 ± 4.900	15.45 ± 4.030	15.62 ± 3.010
	Eosinophils (%)	3.700 ± 0.420	3.870 ± 0.180	3.620 ± 0.370	3.100 ± 0.720
	Basophilic (%)	0.370 ± 0.170	0.220 ± 0.120	0.260 ± 0.110	0.200 ± 0.140
	RBC (×106/µL)	8.520 ± 1.820	8.450 ± 1.170	7.410 ± 0.520	8.750 ± 0.470
	HBG (g/dL)	154.0 ± 6.450	125.0 ± 10.13	99.50 ± 4.930	98.50 ± 11.95
	HCT (%)	80.55 ± 2.590	66.37 ± 6.360	43.85 ± 4.860	44.57 ± 5.140
	MCV (f L)	52.15 ± 4.430	49.55 ± 1.930	58.60 ± 3.330	55.57 ± 2.250
	MCHC (g/dL)	319.0 ± 8.340	335.5 ± 12.04	340.0 ± 18.19	330.7 ± 11.52
	RDW (%)	16.55 ± 0.300	15.37 ± 1.750	14.45 ± 0.380	16.75 ± 1.260
	AST (U/L)	94.50 ± 2.760	105.7 ± 3.72	105.2 ± 4.400	148.9 ± 5.640
	ALT(U/L)	23.18 ± 2.190	42.55 ± 4.780	40.58 ± 2.400	43.35 ± 2.600
	CRE (µmol/L)	39.73 ± 3.140	9.960 ± 0.400	12.14 ± 1.480	15.05 ± 2.670
	URE (mg/dL)	7.810 ± 0.990	43.87 ± 3.070	52.75 ± 3.120	46.40 ± 5.780

^1^ 5D3PC did not show significant alteration in hematological and biochemical parameters in ICR in both sex mice. Values expressed as *n* ± SD. Data are considered statistically significant when *p* < 0.05.

## Data Availability

Not applicable.

## Abbreviations

8-MOP	8-methoxypsoralan
TYR	Tyrosinase
H.Q	hydroquinone
MDA	malondialdehyde
